# Neoadjuvant chemoradiotherapy delivered with helical tomotherapy under daily image guidance for rectal cancer patients: efficacy and safety in a large, multi-institutional series

**DOI:** 10.1007/s00432-019-02881-8

**Published:** 2019-03-04

**Authors:** Berardino De Bari, Alessandra Franzetti-Pellanda, Asma Saidi, Maira Biggiogero, Dieter Hahnloser, Michael Montemurro, Jean Bourhis, Michele Zeverino, Mahmut Ozsahin

**Affiliations:** 1 Radiation Oncology Department, Centre Hospitalier Universitaire Vaudois, CHUV, 46 rue du Bugnon, 1011 Lausanne, Switzerland; 2 Radiation Oncology Department, Centre Hospitalier Régional Universitaire Jean Minjoz, INSERM U1098 EFS/BFC, Besançon, France; 3 Radiation Oncology Department, Clinica Luganese, 6900 Lugano, Switzerland; 4 Surgery Department, Centre Hospitalier Universitaire Vaudois, 1011 Lausanne, Switzerland; 5 Medical Oncology Department, Centre Hospitalier Universitaire Vaudois, 1011 Lausanne, Switzerland; 6 Medical Physics Department, Centre Hospitalier Universitaire Vaudois, 1011 Lausanne, Switzerland

**Keywords:** Helical tomotherapy, Rectal cancer, Image-guided radiotherapy, Toxicity, Neoadjuvant chemoradiotherapy

## Abstract

**Purpose:**

Helical tomotherapy (HT) has been recently introduced in the neoadjuvant treatment of locally advanced rectal cancer. Aim of this study is to report the toxicity and local control rates of a large series of locally advanced rectal cancer patients treated with neoadjuvant chemotherapy and HT under daily image guidance followed by surgery.

**Methods:**

Data from 117 locally advanced rectal cancer patients treated at two Swiss Radiotherapy departments were collected and analyzed. Radiotherapy consisted of 45 Gy (1.8 Gy/fraction, 5 fractions/week delivered in 5 weeks) to the regional pelvic lymph nodes. Seventy patients also received a simultaneous integrated boost (SIB) up to 50 Gy to the tumor and involved nodes (2 Gy/fraction, 5 fractions/week delivered in 5 weeks). Chemotherapy consisted of capecitabine 825 mg/m^2^, twice daily, during the irradiation days. After a median interval of 59 days [95% confidence interval (CI) 53–65 days), all patients underwent surgery.

**Results:**

Median follow-up was 45 months (range 4–90 months). The overall rate of acute grade 2–4 toxicity was 18.8% (*n* = 22). Four patients (3.4%) presented a grade 3 dermatitis (*n* = 1) or diarrhea (*n* = 3), and 1 (0.8%) demonstrated grade 4 rectal toxicity. No patients presented with grade ≥ 3 hematologic toxicity. Six patients (5.1%) had late grade 3 gastrointestinal toxicity. The 4-year local control rate was 88.4% (95% CI 87.5–88.5%).

**Conclusions:**

Neoadjuvant chemoradiotherapy delivered with HT under daily image guidance is well tolerated and shows a high 4-year local control rates.

## Background

Neoadjuvant radiotherapy (RT), with or without chemotherapy, is the standard treatment for patients with locally advanced rectal cancer, as it has been shown to improve the local control of the tumor (Benson et al. 2018). The clinical advantages of chemoradiotherapy (CRT) should be carefully balanced with the significant risk of treatment-related adverse events. In a study from the German Rectal Cancer Study Group, the rates of observed severe acute and late grade 3–4 toxicities were 27% and 14%, respectively (Sauer et al. 2004). In a French study, the overall rate of grade 3–4 toxicities [based on the World Health Organization (WHO) scale] was 14.9%, in which 30% of the patients failed to complete the scheduled treatment (Gerard et al. 2006).

The small bowel is a radiosensitive organ, and the dose delivered to it may at times lead to gastrointestinal toxicity. Several studies have already shown that the risk of both acute and late adverse events is proportional to the maximum prescribed dosage and the total irradiated bowel volume (Baglan et al. 2002; Goupy et al. 2017; Reis et al. 2015). When adopted for the irradiation of pelvic targets, the intensity-modulated radiotherapy (IMRT) has the ability to reduce the volume of the bowel being irradiated, as demonstrated in several dosimetric studies (Guerrero Urbano et al. 2006; Mok et al. 2011), and thereby decreasing the risk of radiation-induced adverse events (Viani et al. 2016; Yu et al. 2015). Despite the above-mentioned evidence, the role of IMRT in the treatment of locally advanced rectal cancer has not yet been deeply studied, and no prospective and/or randomized controlled trial comparing the significance of IMRT with three-dimensional conformal radiotherapy for rectal cancer has been reported. Currently, only 3 studies enrolled more than 70 patients (Teoh and Muirhead 2016; Engels et al. 2014; Hernando-Requejo et al. 2014; Zhu et al. 2014). Four retrospective studies showed that the use of IMRT in the treatment of locally advanced rectal cancer patients could reduce the rates of acute adverse events (Teoh and Muirhead 2016; Jabbour et al. 2012; Parekh et al. 2013; Samuelian et al. 2012).

Recently, helical tomotherapy has been proposed for the treatment of locally advanced rectal cancer. It is performed with a dedicated IMRT machine that allows the delivery of helical volumetric IMRT (HT) and, at the same time, the performance of daily image-guided radiotherapy (IGRT). This combination allows for a more precise definition of the planning target volume (PTV) and accurate irradiation. Dosimetric studies have shown that HT could reduce the doses delivered to the normal tissues in patients affected by lower gastrointestinal cancers (i.e., locally advanced rectal cancer or anal cancers) when compared with conventional 3D- conformal RT (De Bari et al. 2018; Yang et al. 2013) or IMRT (Yeung et al. 2015). Clinical data on the role of HT in the treatment of locally advanced rectal cancer are available, but is mainly in the form of a small study which consisted of only a small cohort of 36 patients (Huang et al. 2014). Despite the potential clinical interest of HT in the treatment of locally advanced rectal cancer, evidence from large patient series is still lacking in the literature.

Therefore, the aim of this study was to report the toxicity profile and the clinical outcomes of 117 locally advanced rectal cancer patients treated with neoadjuvant long-course CRT delivered with HT and under the daily image guidance at two Swiss Radiation Oncology Departments.

## Patients and methods

### Study population

In this study, we included adult patients (≥ 18 years) with a histological diagnosis of locally advanced rectal cancer [cT3–T4 and/or cN0-2 disease, staged according to the 7th edition of the Union Internationale Contre le Cancer (UICC) staging system] treated with curative neoadjuvant CRT at the Radiation Oncology departments of the Centre Hospitalier Universitaire Vaudois (CHUV) and the Clinica Luganese (CL) between January 2010 and December 2015. Before treatment, all patients underwent a total body computed tomography (CT) scan for locating any systemic disease and a pelvic magnetic resonance imaging (MRI) for assessing the status of the tumor and regional lymph nodes.

### Population and treatment

A total of 117 patients (84 men and 33 women) with a histological diagnosis of locally advanced rectal cancer located within 15 cm from the anal verge were admitted to the Radiation Oncology Department of the CHUV (*n* = 70) and CL (*n* = 47), and were treated with long-course neoadjuvant CRT. The median age of the patients was 65 years (range 32–85 years). The clinical features of the patients treated at the two centers had no significant differences. Table 1 summarizes their clinical features and treatment details.

**Table 1:** Clinical and therapeutic features of the 117 patients with locally advanced rectal cancer

Characteristic	No. of patients (%)
Gender	
Male	84 (71.8)
Female	33 (28.2)
Tumor location	
Low rectum	67 (57.2)
Middle rectum	47 (40.2)
High rectum	3 (2.6)
Clinical tumor stage	
cT2	11 (9.4)
cT3	96 (82)
cT4	10 (8.6)
Clinical nodal stage	
cN0	35 (30)
cN1	61 (52)
cN2	21 (18)
Tumor stage^a^	
IIA	34 (29.1)
IIB	1 (0.8)
IIIA	9 (7.7)
IIIB	60 (51.3)
IIIC	13 (11.1)
Tumor differentiation	
Well	20 (17.1)
Moderate	72 (61.5)
Poor	8 (6.8)
Not available	17 (14.6)
Radiotherapy	
Radiotherapy alone	1 (0.8)
Chemoradiotherapy	116 (99.2)
Treated without SIB	47 (40.1)
Treated with SIB	70 (59.9)
Surgery	
Abdominal-perineal resection	23 (19.7)
Low anterior resection	94 (80.3)

*SIB* simultaneous integrated boost

^a^Defined using the 7th edition of the Union Internationale Contre le Cancer **(**UICC) staging system

All but one patient received concomitant neoadjuvant CRT. The patient who refused concomitant chemotherapy received radiotherapy alone. All patients received preoperative treatment without interruptions for a median treatment duration of 36 days (range 30–67 days). After a median interval of 59 days (95% CI 53–65 days), all patients underwent surgery with total mesorectal resection. Ninety-four patients (80.3%) received a low anterior resection, whereas 23 (19.7%) received an abdominoperineal resection. The resection status was classified as R0 in 107 patients and R1 in 3 patients, and was not reported in 7 patients. The median number of removed nodes was 15 (range 0–47). Table 2 summarizes the pathological data relating to the surgical specimens.

**Table 2:** Pathological data of the surgical specimens from the 117 patients with locally advanced rectal cancer

Feature	Number of patients (%)
Tumor location^a^	
Low rectum	46 (39.3)
Middle rectum	45 (38.5)
High rectum	26 (22.2)
Pathological tumor stage^b^	
ypT0	17 (14.5)
ypT1	16 (13.7)
ypT2	32 (27.3)
ypT3	45 (38.5)
ypT4	7 (6.0)
Pathological nodal stage	
ypNx	1 (0.9)
ypN0	92 (78.6)
ypN1a	3 (2.6)
ypN1b	8 (6.8)
ypN1c	2 (1.7)
ypN2a	8 (6.8)
ypN2b	3 (2.6)
Tumor regression grade (TRG)^c^	
TRG 1	17 (16.8)
TRG 2	35 (34.7)
TRG 3	20 (19.8)
TRG 4	3 (3)
TRG 5	26 (25.7)
Tumor and nodal staging differences before and after neoadjuvant treatment	
Tumor downstaging	65 (55.6)
cT2 → ypT0	2 (3.1)
cT3 → ypT0	13 (20)
cT3 → ypT1	15 (23.1)
cT3 → ypT2	28 (43.1)
cT4 → ypT0	1 (1.5)
cT4 → ypT2	1 (1.5)
cT4 → ypT3	5 (7.7)
Tumor upstaging	7 (6.0)
cT2 → ypT3	3 (42.9)
cT3 → ypT4	4 (57.1)
Nodal downstaging	64 (54.7)
cN1 → ypN0	46 (71.9)
cN2 → ypN0	15 (23.4)
cN2 → ypN1a-b	3 (4.7)
Nodal upstaging	11 (9.4)
cN0 → ypN1a–c	3 (27.3)
cN0 → ypN2b	1 (9.1)
cN1 → ypN2a	4 (36.3)
cN1 → ypN2b	3 (27.3)

^a^Tumor location was reassessed during pathologic examination

^b^Defined using the 7th edition of the Union Internationale Contre le Cancer **(**UICC) staging system

^c^Data are available for 101 patients. TGR was evaluated using the Mandard score (Passoni et al. 2013)

Of the 117 patients, 62 (52.9%) received adjuvant chemotherapy, 47 (40.1%) did not receive it, and for 8 patients (7%), this information was unknown, as they were followed in other centers. 17/62 patients (27.4%) received 4–12 cycles of adjuvant capecitabine or intravenous (i.v) 5-fluorouracile (5-FU) alone, 43 patients (69.3%) received 4–12 cycles of oxaliplatin with capecitabine or i.v. 5-FU, and 2/62 (3.2%) patients had other chemotherapy regimens. The number of cycles was decided by the medical oncologist depending on the final pathologic data. The median number of cycles was 6 (range 4–12).

### Ethics, consent, and permissions

This study was approved by the Ethics Committees of CHUV and CL. According to the local Federal rules, this approval is also an approval of consent for patients’ participation in this study.

### Neoadjuvant chemoradiotherapy (CRT) details

All but one patient were treated with concomitant oral capecitabine (Roche, Basel, Switzerland) (825 mg/m^2^, twice daily, 5 days/week) during the irradiation days. Adjuvant chemotherapy was prescribed by abiding to the National Comprehensive Cancer Network (NCCN) guidelines (1) and was delivered to patients who had a performance status ≤ 2 based on the Eastern Cooperative Oncology Group (ECOG) score. None of the patients received neoadjuvant chemotherapy alone.

After performing a continuous simulation CT with a 2-mm slice thickness of the abdomen (all patients were simulated with a full bladder), a treatment plan was computed with the Tomotherapy Hi-Art II System (Accuray, canton of Vaud, Switzerland). Of note, HT was delivered to all of the enrolled patients.

Treatment volumes were delineated after the primary tumor and involved regional lymph nodes were identified by digital rectal examination, endoscopy, or MRI ± positron-emission tomography/CT (PET/CT). Two clinical target volumes (CTVs) were defined. The CTV1 encompassed the pelvis, including the gross target volume of the primary tumor (GTV-T) and the involved lymph nodes (if any, GTV-N), comprising of the entire mesorectum, pre-sacral, internal iliac, and obturator lymph nodes. External iliac nodes were part of the CTV1 in T4 patients. The Radiation Therapy Oncology Group (RTOG) consensus atlas (https://www.rtog.org/CoreLab/ContouringAtlases/Anorectal.aspx) was adopted for the definition of the CTV1 (Myerson et al. 2009). The planning target volume (PTV1) was defined as the CTV1 with a margin of 5 mm and received 45 Gy of radiation (1.8 Gy/fraction, 5 fractions/week, delivered in 5 weeks), and prescribed to the isocenter of the tumor. The local protocols of the CHUV Radiation Oncology Department envisaged also a second volume (CTV2), consisting of the GTV-T with the corresponding mesorectum at a margin of 2 cm in the craniocaudal direction and the GTV-N (if any) with an isotropic margin of 5 mm. The PTV2 was defined as the CTV2 with a margin of 5 mm and received 50 Gy of radiation (2 Gy/fraction, 5 fractions/week, delivered in 5 weeks), prescribed to the isocenter and delivered simultaneously using the simultaneous integrated boost (SIB) technique. Treatment plans were created for the TomoHDA system (Accuray, Sunnyvale, USA) with TomoEdgeTM option. They were generated using the convolution/superposition algorithm (Version 5.1.0.4, Accuray, Sunnyvale, USA) with a dose-grid size of 2.2 × 2.2 × 3 mm^3^ and designed with a field width of 2.5 cm, a pitch of 0.287, and a modulation factor between 2 and 3.

The estimated organs at risk (OARs) were the bowel (defined as abdominal cavity), the bladder (contoured as full bladder), and the femoral heads. All the OARs were contoured from the mid third lumbar vertebrae to the lowest extent in the pelvis.

The dosimetric goals were to deliver at least 98% of the prescribed radiation dose to at least 98% of the PTV and a maximum dose to the PTV of 107% of the prescribed dose while minimizing the volume of the irradiated bowel. Doses to more than 250 cm^3^, 195 cm^3^, and 50 cm^3^ of the bowel were kept to < 35 Gy, < 40 Gy, and < 50 Gy, respectively. Dose constraints for the femoral heads were 45 Gy to less than 10% and maximum dose of 50 Gy. Considering the total doses that were delivered, no special constraints were applied for the bladder, but the dose to the bladder was kept as low as reasonably achievable.

Before each fraction of irradiation, the patients underwent a fan-beam MV-CT using the modality integrated in the HT. The setup of the patient was adjusted daily if the setup exceeded the CTV-to-PTV margin of 5 mm. During the CRT treatment, all patients received a weekly clinical evaluation to record any acute adverse events.

### Study endpoints

Data were collected using hospital electronic records and, if necessary, were updated until the end of this analysis. After the end of the treatment, the enrolled patients had routine follow-up visits every 3 months in the first 2 years, then every 6 months afterwards.

Acute and late toxicities were retrospectively evaluated using the National Cancer Institute Common Toxicity Criteria for Adverse Events (NCI-CTCAE), version 4.0. In some cases, when the description of the toxicity was not clear, the worst case scenario was considered (i.e., in the case in which it was not possible to clearly state whether the patient presented with a grade 1 or 2 toxicity, it was recorded as a grade 2 toxicity in our database). Toxicity recorded within 3 months after the end of the treatment was considered as acute toxicity, whereas all others observed afterwards were considered as late toxicities. In each of the involved center, only one of the co-authors performed the retrospective scoring of toxicities to reduce inter-observer variability (BDB for the CHUV cases and AFP for the CL cases).

Local control (LC), overall survival (OS), disease-free survival (DFS), and distant metastases-free survival (DMFS) rates were the secondary endpoints of this study and were calculated from the date of diagnosis to the date of local relapse, death from any cause, death from any relapse, and death from diagnosis of distant metastases, respectively. The date of deaths was confirmed based on the death certificates provided by the patients’ relatives. Pathological complete response (pCR) was defined according to the tumor regression grade (TRG), and the tumor (T) and nodal (N) stages were defined according to the results of pathological examination. The TRG was evaluated using the Mandard regression score (Mandard et al. 1994).

### Statistical analysis

The Fisher’s exact test for values ≤ 5 or Chi-square test for values > 5 was used to compare categorical variables. Kaplan–Meier method was adopted to estimate the survival curves, and the log-rank test was used to calculate the differences in survival. Patients with the events or lost at follow-up were censored. Assuming a normal distribution, confidence intervals (CIs) were computed from standard errors using the formula *k* × SE, with *k* = 1.96 for 95% CI. The Statistical Package for the Social Sciences, version 18.0 (SPSS Inc., Chicago, IL, USA) was used for statistical calculations.

## Results

### Acute toxicities

All patients were assessed for acute toxicities (Table 3). In all, there were 22 patients (18.8%) who presented with a grade 2–4 toxicity, with some of them presenting a toxicity of more than only one organ. There were four patients (3.4%) who had to be re-hospitalized (duration of hospitalization, 8 days), as they presented with grade 3 dermatitis (*n* = 1) and/or diarrhea (*n* = 3). There was only 1 (0.8%) observed case of grade 4 rectal toxicity (proctitis). No patients presented grade ≥ 3 hematologic toxicity.

**Table 3:** Acute and late toxicities of neoadjuvant concomitant chemoradiotherapy for the 117 patients with locally advanced rectal cancer

Adverse events	Grade (cases)					
	0	1	2	3	4	Not reported
Acute						
Dermatitis	43	55	16	1	0	2
Diarrhea	43	58	12	3	0	1
Proctitis	110	7	0	0	0	0
Cystitis	104	12	1	0	0	0
Rectal/abdominal pain	97	18	0	1	1	0
Late						
Intestinal/rectal	95	20	0	1	1	0
Urinary	115	2	0	0	0	0
Fecal incontinence^a^	57	1	3	2	2	17

6/117 patients (5.7%) presented a Grade 3 surgery related ileus and fistula

Some patients presented several adverse events. For instance, if one patient had grade 2 dermatitis and diarrhea, accompanied with grade 4 rectal pain, this was considered as one case for each toxicity

^a^Evaluated in 82 patients who were colostomy-free at the last follow-up

### Late toxicities

All patients were followed for at least 3 months, with a median of 45 months (range 4–90 months). Six patients (5.1%) presented with grade 3–4 late toxicity (Table 3). One patient presented with treatment-induced bowel adhesions and chronic diarrhea, which required several surgical interventions in our or other hospitals. As note, that patient did not present with any particular bowel toxicity during the treatment and had pre-sacral relapse 14 months after the diagnosis.

### Pathological response

Data on TRG were available for 101 (86.3%) patients (Table 2). Tumor regression (TRG 1–4) was reported in 75/101 (74.2%) patients. Seventeen (16.8%) of the 101 patients presented a pCR.

Tumor and nodal downstaging was observed in 65/117 (55.6%) and 64/117 (54.7%) patients (Table 2). The proportion of patients with a TRG 1 or with a TRG = 1–2 was higher in the SIB group than in the non-SIB group  [TRG1 = 12/70 (17.1%) patients in the SIB group vs. 2/47 (4.2%) patients in the non-SIB group, *p* = 0.017; TRG 1–2 = 33/70 patients in the SIB group vs. 10/47 patients in the non-SIB group, *p* < 0.001, respectively]. We also evaluated whether the interval between the end of radiotherapy and surgery, evaluated at 8 and 12 weeks and at 53 days (median of the population with a known TRG value), could influence the possibility of obtaining a TRG = 1–2, but no significant correlations were found.

### Clinical outcomes

**Fig. 1:**
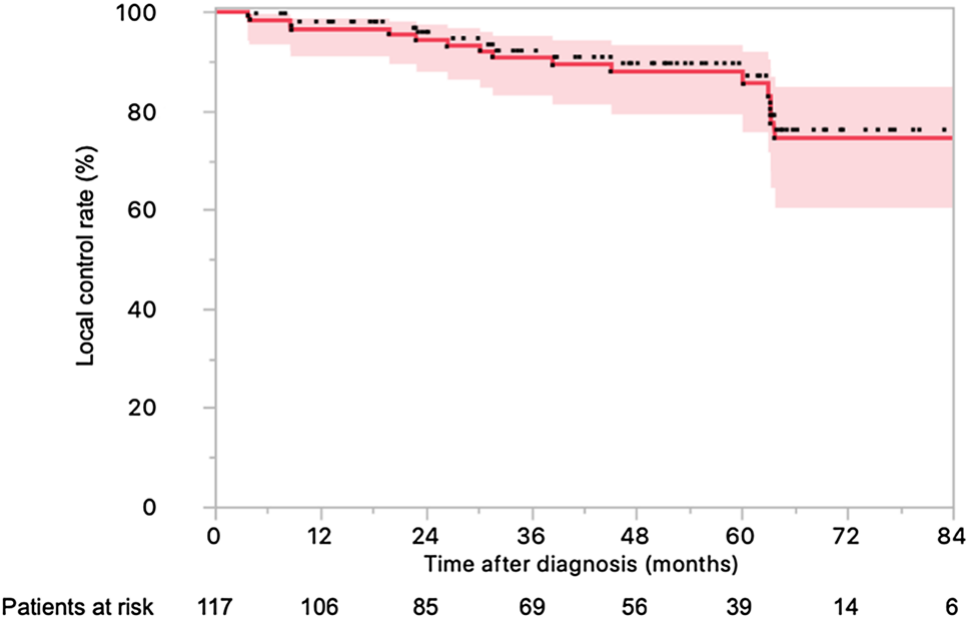
Local control in 117 locally advanced rectal cancer patients treated with neoadjuvant chemotherapy and helical tomotherapy with daily image guidance followed by surgery (with the 95% confidence interval)

**Fig. 2:**
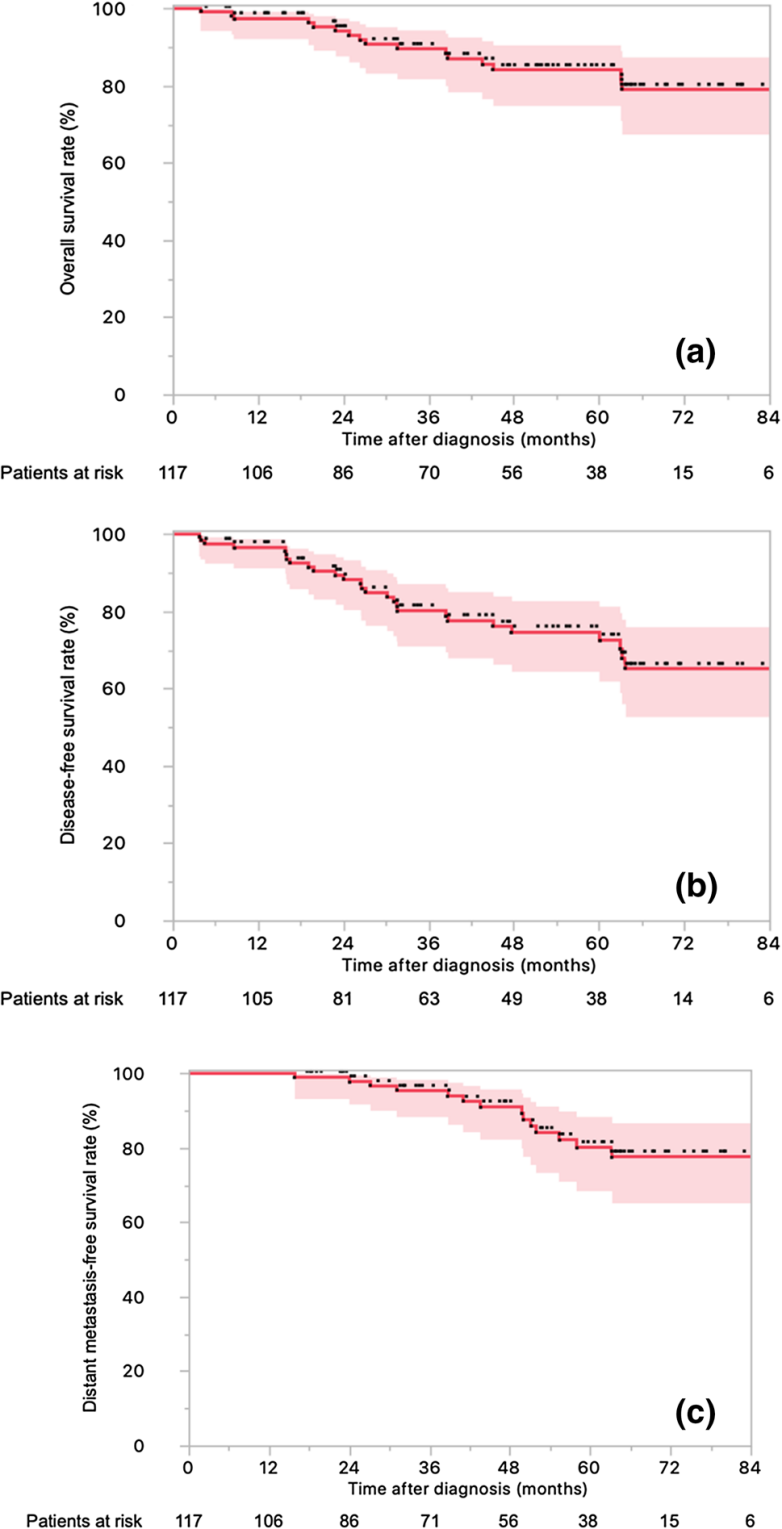
Kaplan–Meier survival curves of 117 locally advanced rectal cancer patients treated with neoadjuvant chemotherapy and helical tomotherapy with daily image guidance followed by surgery. **a** Overall survival rate. **b** Disease-free survival rate. **c** Distant metastases-free survival rate (with the respective 95% confidence intervals)

Sixteen patients presented local relapse, 14 had distant relapse, and 16 died. The actuarial 4-year LC (Fig. 1), OS (Fig. 2a), DFS (Fig. 2b), and DMFS (Fig. 2c) rates were 88.4% (95% CI 87.5–88.5%), 84.2% (95% CI 83.1–85.3%), 74.6% (95% CI 73.0–75.0%), and 91.2% (95% CI 90.1–92.2%).

## Discussion

In this large series of locally advanced rectal cancer patients treated with neoadjuvant concomitant CRT delivered with HT under daily image guidance, our treatment approach demonstrated low rates of severe acute and late toxicities, with a high 4-year LC rate of 88.4%. A rough comparison of our results with the historical series of patients treated in the context of randomized trials, usually using 2D- or conformal 3D-RT techniques (Sauer et al. 2004; Gerard et al. 2006), showed a clear reduction in the rates of acute and late non-hematological toxicities. Consequently, this translates into an improvement of the patients’ compliance to CRT delivered with HT.

The adoption of IMRT in the treatment of locally advanced rectal cancer is an attractive option because of its potential to reduce the doses administered to the bowel and the risk of acute and late gastrointestinal toxicities. In a dosimetric study on five patients, Guerrero Urbano et al. (2006) compared 3D-RT and several IMRT plans. In their study, IMRT showed to have a better PTV coverage and a significant reduction of radiation volumes to the bowel. Similar results were reported by Mok et al. (2011) in a dosimetric study on ten patients in which they observed that when compared with 3D-RT, IMRT demonstrated a superior PTV coverage, dose homogeneity and conformality, and also achieved a concomitant reduction in radiation doses to the bowel. These dosimetric evidences support the adoption of IMRT in this clinical setting for its potential to reduce bowel toxicity.

From a clinical point of view, no prospective trials had tested the impact of IMRT on acute and late toxicities of neoadjuvant CRT for locally advanced rectal cancer, but there were four retrospective studies showing the potential of IMRT in reducing the toxicity in this setting (Jabbour et al. 2012; Parekh et al. 2013; Samuelian et al. 2012; Yang et al. 2013). Jabbour et al. (2012) analyzed 86 patients with rectal cancer treated with preoperative IMRT (*n* = 30) and 3D-RT (*n* = 56). Patients in the IMRT group had a significant reduction in re-hospitalization rate and visits to the emergency services (*p* = 0.005), no treatment interruptions (*p* < 0.001), and a significant reduction in grade ≥ 3 toxicities as compared to grade ≤ 2 toxicities (*p* = 0.0016). The rates of grade ≥ 3 diarrhea were 3% in the IMRT group and 9% in the 3D-RT group (*p* = 0.31). Parekh et al. (2013) compared 28 locally advanced rectal cancer patients treated with 3D-RT and 20 patients treated with IMRT and observed a significant reduction in grade ≥ 2 gastrointestinal toxicity (60.7% vs. 30%, *p* = 0.036) and grade ≥ 2 diarrhea (42.8% vs. 10%, *p* = 0.014) in the IMRT group. Samuelian et al. (2012) compared treatment efficacy of 61 locally advanced rectal cancer patients treated with 3D-RT with 31 patients treated with IMRT. They observed that for patients treated with IMRT, there were 23% and 10% who presented grade 2 diarrhea and enteritis, respectively. These rates were significantly lower than those of patients treated with 3D-RT, which were 48% (*p* = 0.02) and 30% (*p* = 0.015), respectively. Yang et al. (2013) reported the largest study on this issue. They compared 98 patients treated with IMRT and 79 treated with 3D-RT. In their multivariate analyses, female gender and the use of 3D-RT were the most predictive factors for grade ≥ 2 diarrhea [area under the curve (AUC) = 0.76, *p* < 0.001]. A higher rate of grade ≥ 2 diarrhea was observed in the 3D-RT group during the treatment (22% vs. 12%, *p* = 0.03) and at week-5 after the end of RT (32% vs. 11%, *p* = 0.001). Our results, which had an overall rate of 18.8% for grade ≥ 2 toxicity, were comparable with the results reported in these aforementioned retrospective studies.

HT is a more complex form of IMRT, and it also allows a daily verification of the setup for the patient, thus permitting a reduction of the CTV-to-PTV margins. In the retrospective studies cited above (Jabbour et al. 2012; Parekh et al. 2013; Samuelian et al. 2012), a uniform margin of 1 cm was used when daily image guidance was not performed and 0.5 cm when daily image guidance was adopted.

In one retrospective study reporting on 36 locally advanced rectal cancer patients treated with neoadjuvant HT (Huang et al. 2014), the investigators found that for all patients who received 45 Gy of irradiation to the regional nodes and a SIB of up to 50.4 Gy to the tumor (5 fractions/week over 5 weeks), in addition to concomitant capecitabine (850 mg/m^2^, twice daily, during irradiation days) for a median follow-up of 35 months, they had a rate of grade ≥ 3 toxicities of 11.1%, which was higher than that in the present study. The most common grade 3 late toxicities in our study were ileus and fistula, which was reported in 5.7% of the patients. In addition, worthy to mention that despite a daily control of the setup of their enrolled patients, the margins adopted in the study by Huang et al. (2014) were larger than those adopted in the present study, as the CTV_45Gy_ was expanded to 1 cm, while 0.5 cm were added to the smallest CTV_50.4Gy_, to obtain the respective PTVs. These larger margins applied to the largest CTV could explain the higher rate of grade ≥ 3 toxicity (11.1%) as compared with the rate in our series (4%). It is likely that the volume of small bowel receiving higher doses was greater in their study, thus explaining their higher rate of toxicity. Passoni et al. (2013) investigated the feasibility of preoperative adaptive CRT using HT by delivering a concomitant boost to the residual tumor during the last 6 fractions of irradiation after a new simulation CT scan and pelvic MRI in 25 enrolled patients. The investigators delivered 41.4 Gy in 18 fractions (2.3 Gy/fraction) to the tumor and regional nodes. After 9 fractions, simulation CT scan and MRI were repeated to re-plan new volumes that were used in the last 6 fractions as the volume for a boost of 3.0 Gy per fraction (total dose: 45.6 Gy in 18 fractions), while concomitantly delivering 2.3 Gy per fraction to PTV outside these new volumes. The authors reported 2 cases of grade 3 acute diarrhea (8%) and 1 case of grade 3 acute proctitis (4%). No data on late toxicity were reported. Engels et al. (2014) reported the results of 102 patients treated with CRT using HT. All patients received 46 Gy of irradiation to the pelvis, and there were 57 patients presenting with a narrow circumferential resection margin (CRM) who received a SIB of up to 55.2 Gy (2.4 Gy/fraction). The CTV-to-PTV margins were 0.7–1.0 cm for the CTV_46Gy_ and 0.5 cm for the CTV_55.2Gy_. After a median follow-up of 54 months, the authors reported 37 cases (36.3%) of grade ≥ 2 and 9 cases (8.8%) of grade ≥ 3 late gastrointestinal toxicity (with two cases of grade 5 enteritis), as well as 24 cases (23.5%) of grade ≥ 2 genitourinary toxicity. There reported rate of pCR was 8%.

Furthermore, SIB is usually adopted in studies reporting the use of HT in the treatment of locally advanced rectal cancer. In the present study, it was adopted in 60% of the patients, at dose levels which are comparable to those delivered in the study by Huang et al. (2014). These authors reported a pCR rate of 14.3% (assessed with the Dworak score, corresponding to a Mandard score = 1) and major response (corresponding to a Mandard score = 1 or 2) rate of 60%. In the present study, the rates of patients treated with SIB who presented a TRG score = 1 or 1–2 were 17.1% and 47.1%, which are comparable to the rates reported by Huang et al. (2014), and significantly higher when compared with the rates of patients treated without SIB who presented a TRG = 1 or 1–2 (4% and 21%). In the study by Passoni et al. (2013), delivering a total dose of 45.6 Gy in 18 fractions (2.5 Gy/fraction), the rate of pCR was 30%. The interest of the SIB is that improves the dosimetry of the treatment plan, could shorten the treatment time and it could allow the reduction of the toxicity of the treatments. All these data on the SIB in the treatment of rectal cancer patients confirm the interest of this approach also in this clinical setting, as it has been already shown in other tumor sites (Lupattelli et al. 2017; Franco et al. 2014, 2015, 2016, 2018; But-Hadzic et al. 2016; De Rose et al. 2018; Chatterjee et al. 2019; Magli et al. 2018).

Although the present study provided some interesting data on the potential of HT in the treatment of locally advanced rectal cancer, there are some drawbacks that need to be addressed, such as the retrospective non-randomized design, the multi-institutional nature of the data and the lack of stratification for toxicity according to the prescribed RT dose, chemotherapy schedule, and the distance between the distal end of the tumor the anal margin. Moreover, data on acute and late toxicities were collected from hospital records, as usually done in retrospective studies, and were retrospectively re-scored. Even if the grade of the toxicity was “up-graded” in doubtful cases, this may have possibly resulted in an underestimation of the real data. This cohort is heterogeneous, with 70 patients received SIB, making the interpretation of the findings somehow challenging.

Nevertheless, the introduction of a new technique of RT should always be driven by clinical criteria, as the reduction of toxicity and/or the improvement of outcomes. In the present study, the significantly higher rate of pCR in the group of patients who received higher doses of RT using SIB confirms the potential role of dose escalation in locally advanced rectal cancer and deserves further prospective, randomized, investigations to confirm the findings of this study. Given the prognostic value of pCR in locally advance rectal cancer (Benson et al. 2018), and the importance of increasing the dose to safely increase the rate of pCR, there is an interest and potential of using HT to treat these patients. Moreover, low rates of mild and severe toxicities and a high 4-year LC rate show that smaller margins could be added to the CTV when daily image guidance is performed. This more conservative way to define target volumes seems to improve the tolerance of the patients; without affecting LC.

## Conclusions

The present study showed that CRT delivered with HT under daily image guidance is safe, with low rates of acute and late severe toxicity. TRG was improved when using SIB. The 4-year LC rate is encouraging and should be confirmed after long-term follow-up. We propose that highly conformal techniques under daily image guidance could be the standard of care in the neoadjuvant treatment of locally advanced rectal cancer patients.

## Data Availability

The data sets generated and/or analyzed during the current study are not publicly available due the Swiss privacy policy but are available from the corresponding author on reasonable request.

## Abbreviations

3D-RT: 3-Dimensional radiotherapy
5-FU: 5-Fluorouracile
CHUV: Centre Hospitalier Universitaire Vaudois
CI: Confidence interval
CL: Clinica Luganese
CRM: Circumferential resection margin
CRT: Chemoradiotherapy
CT: Computed tomography
CTV: Clinical target volume
DFS: Disease-free survival
ECOG: Eastern Cooperative Oncology Group
G: Grade
GTV: Gross target volume
HT: Helical tomotherapy
IGRT: Image-guided radiotherapy
IMRT: Intensity-modulated radiotherapy
LC: Local control
M: Metastases
MFS: Metastases-free survival
MRI: Magnetic resonance imaging
MV: MegaVolt
N: Nodes
NCCN: National comprehensive cancer network
NCI-CTCAE: National Cancer Institute-Common Toxicity Criteriafor Advers Events
OARs: Organs at risk
OS: Overall survival
pCR: Pathological complete response
PET/CT: Positron-emission tomography/CT
PTV: Planning target volume
RT: Radiotherapy
RTOG: Radiation Therapy Oncology Group
SIB: Simultaneous integrated boost
SPSS: Statistical package for the social sciences
T: Tumor
TRG: Tumor regression grade
UICC: Union Internationale Contre le Cancer
WHO: World Health Organization
